# Notes and News

**Published:** 1905-12-30

**Authors:** 


### Notes and News.

The secretaryship at the Royal Portsmouth Hospital has been filled by the election of Mr. B. Wagstaff, who received his training at the Mount Vernon Hospital.

After several meetings and a good deal of controversy, it has been decided to extend the term of office of the present Brighton Hospitals Board from December 31 to March 31, Colonel Somers Clarke acting as chairman.

Mr. J. Stephen Neil, Secretary of the Royal Portsmouth Hospital, has been appointed House Governor of Wolverhampton General Hospital. Mr. Neil was formerly connected with the Great Northern Central Hospital.

At a meeting of the Selby Cottage Hospital last Saturday an offer was received from Miss Standering, of Selby, to defray the cost of building a surgical ward for women and an operation-room for the hospital. Miss Standering also proposed to give £2,000 towards the endowment of the institution, an offer which was gratefully accepted by the board of management.

Birmingham charities and public institutions benefit to the extent of over £80,000 by the will of the late Mr. John Feeney. Among other benefactions are £20,000 to the University of Birmingham, £10,000 to the Birmingham General Hospital, £1,000 to the Queen's Hospital, £1,000 to the Birmingham General Dispensary, £1,000 to the Birmingham and Midland Eye Hospital, £1,000 to the Birmingham and Midland Hospital for Women, £1,000 to the Free Hospital for Sick Children, £1,000 to the General Institution for the Blind. The Wolverhampton and Staffordshire General Hospital and the Coventry and Warwickshire Hospital each receive £1,000.

The Kent and Canterbury Hospital has just undergone thorough restoration and partial rebuilding at a cost of £13,000. In the old door of the operating theatre, now removed, were the rings through which were passed the cords for securing patients during operations before the discovery and introduction of anæsthetics.

Dr. Frederick Ensor, who died at Upwey, Dorset, the other day, was for many years in the Government medical service in Port Elizabeth. He was a personal friend of Dr. Livingstone and accompanied him on several of his journeys. During his service at Port Elizabeth, Dr. Ensor was called in to examine the body of the informer Carey on his assassination, and as no one else would perform the task he read the Burial Service over him.

Miss Matilda Mercer, of Clifton, who died on September 21, bequeathed £1,000 to the Bristol Royal Infirmary, £1,000 to the Bristol General Hospital, £500 to the Bristol Royal Hospital for Sick Children and Women, £200 to the Bristol Asylum for the Blind, and £200 to the Clifton Dispensary. Subject to the life interest of her sister, she also left £4,000 to the Bristol Royal Infirmary, £4,000 to the Bristol General Hospital, £3,000 to the Queen Victoria Jubilee Convalescent Home, Durdham Down, £1,500 to the Bristol Royal Hospital for Sick Children and Women, for the specific purpose of endowing two beds in each of these institutions to be called the "John Mercer" and "Matilda Mercer" beds. In addition she left £1,000 to the West of England Sanatorium at Weston-Super-Mare, £1,000 to the Bristol Eye Hospital, £1,000 to the Orthopædic Hospital, £1,000 to the Home for Crippled Children, Bristol, £500 to the Bristol Asylum for the Blind, and £500 to the Clifton Dispensary.

Many interesting matters relative to epidemiology and the treatment of the sick are to be found in the annual Blue-book, just issued, on Sanitary Measures in India (1903-1904). It appears that in 1903 there were 2,500 Government hospitals and dispensaries, where no fewer than 372,857 in-patients and 22,399,350 out-patients were treated—truly astonishing totals, which are excellent testimonials to the beneficence of British rule. In the section dealing with the army in India some notable differences are apparent between the British and native troops as to their liability to various diseases. Among the British troops the chief causes of sickness were, as usual, malaria and venereal diseases, which accounted for 23.9 and 23.5 per cent. respectively of all admissions to hospital. But these diseases were not very fatal; and the chief cause of mortality was enteric fever, which was responsible for 32 per cent. of deaths from all causes in the European army, while hepatic abscess caused 10 per cent. In the native army, on the other hand, pneumonia was the most frequent cause of mortality, accounting for 30 per cent. of all the deaths, while remittent fever accounted for 9 per cent. It is a noteworthy fact that among the native troops the admission rate for venereal diseases was only one-tenth of that among Europeans.

